# PD-1/PD-L1抑制剂联合治疗相关不良反应的研究进展

**DOI:** 10.3779/j.issn.1009-3419.2021.101.26

**Published:** 2021-07-20

**Authors:** 晓玉 郭, 倜 温, 秀娟 曲

**Affiliations:** 110001 沈阳，中国医科大学附属第一医院肿瘤内科 Department of Medical Oncology, the First Hospital of China Medical University, Shenyang 110001, China

**Keywords:** 肿瘤, PD-1/PD-L1抑制剂, 联合治疗, 不良反应, Tumor, PD-1/PD-L1 inhibitors, Combination therapy, Adverse events

## Abstract

免疫检查点抑制剂（immune checkpoint inhibitors, ICIs）的发展显著改善了肿瘤患者的预后，但仍然有大部分的无效和获得性耐药人群。为了提高临床获益，已有多项研究对ICIs联合治疗展开了积极探索，并取得了良好的治疗效果。随着ICIs联合治疗在临床中的逐步应用，ICIs联合治疗的安全性及相关不良反应的管理也越来越受关注。本文论述了以程序性细胞死亡蛋白1/配体1（programmed cell death protein 1/ligand 1, PD-1/PD-L1）抑制剂为代表的ICIs联合治疗相关不良反应的特点，以期为临床实践中ICIs联合治疗前评估相关毒性、制定个体化治疗策略提供参考。

## Abstract (en)

The development of immune checkpoint inhibitors (ICIs) has significantly improved the prognosis of cancer patients, but a large population of patients are still ineffective to ICIs treatment or develop aquired drug resistance. In order to improve the clinical benefits, a number of studies on ICIs based combination therapy have been actively explored, and have achieved satisfactory results. With the application of ICIs based combination therapy in clinical practice, increasing attention has been paid to the safety of combination therapy and the management of treatment-related adverse events. In this review, the characteristics of adverse events related to ICIs based combination therapies, especially programmed cell death protein 1/protein ligand 1 (PD-1/PD-L1) inhibitors are discussed, in order to provide profound thoughts for toxicity evaluation and individualized treatment decision in future clinical practice.

免疫检查点抑制剂（immune checkpoint inhibitors, ICIs）的发展改变了肿瘤的全身治疗方法。ICIs在多种类型肿瘤治疗中的客观应答率高、应答持久，已获批应用于多个瘤种的治疗，包括恶性黑色素瘤^[1]^、非小细胞肺癌（non-small cell lung cancer, NSCLC）^[2]^、小细胞肺癌^[3]^和尿路上皮癌^[4]^等。尽管ICIs治疗可以使部分人群获得持久获益，但仍然有大部分的无效和获得性耐药人群，而ICIs联合治疗是提高抗肿瘤免疫反应、克服不同机制耐药的可行策略之一。目前，不同ICIs联合，以程序性细胞死亡蛋白1/配体1（programmed cell death protein 1/ligand 1, PD-1/PD-L1）抑制剂为代表的ICIs与化疗、抗血管生成药物、放疗等的联合治疗已在多种实体瘤中观察到更好的临床获益，并被批准应用于多种肿瘤的临床治疗。

ICIs通过激活T细胞并促进免疫系统识别和攻击癌细胞的能力，同时也激活自身免疫系统，导致机体一些正常细胞也受到免疫系统的攻击产生免疫相关不良反应（immune-related adverse events, irAEs）。ICIs单一治疗总体安全，不同类型ICIs的毒性不同。细胞毒性T淋巴细胞相关抗原4（cytotoxic T lymphocyte-associated antigen 4, CTLA-4）抑制剂总体irAEs发生率较PD-1/PD-L1抑制剂高^[5]^，尤其是腹泻、结肠炎、皮疹发生率明显增加^[6]^。PD-1抑制剂与PD-L1抑制剂总体irAEs发生率相似^[5]^，PD-1抑制剂较PD-L1抑制剂肺炎发生率增加^[7]^。ICIs不同剂量及时间间隔的irAEs发生率也不相同^[8]^。

目前，临床医生对单一ICIs的irAEs管理已经积累了一些经验，但对于irAEs发生的病理机制仍然处在探索阶段，可能的机制为ICIs通过解除免疫抑制、活化T细胞功能增强免疫，通过提高T细胞对正常组织表达的自身抗原的应答，增加自身免疫抗体的表达，增加免疫因子的分泌和释放等诱发自身免疫炎症。ICIs联合治疗的作用机制更加复杂，联合治疗可能会改变单一ICIs治疗带来的免疫增强作用，进而影响治疗相关不良发应（treatment-related adverse events, TRAEs）及irAEs的类型和严重程度。随着ICIs联合治疗在临床实践中的逐步应用，联合治疗的安全性及相关不良反应的管理也越来越受关注。本文总结了以PD-1/PD-L1抑制剂为代表的ICIs与CTLA-4抑制剂、化疗、抗血管内皮生长因子受体（vascular endothelial growth factor receptor, VEGFR）单克隆抗体、小分子酪氨酸激酶抑制剂（tyrosine kinase inhibitors, TKIs）、放疗联合的相关不良反应特点，以期为临床实践中PD-1/PD-L1联合治疗前评估相关毒性、制定个体化治疗策略提供参考。

## 1 PD-1/PD-L1抑制剂联合CTLA-4抑制剂

PD-1/PD-L1抑制剂与CTLA-4抑制剂作用机制不同，在调节适应性免疫中起互补作用。临床前模型显示，联合阻断PD-1和CTLA-4可获得更强的抗肿瘤活性^[9]^。

### 1.1 PD-1抑制剂联合CTLA-4抑制剂

纳武利尤单抗联合伊匹木单抗首先在转移性黑色素瘤中表现出良好的疗效，但不良反应也较单一ICIs治疗增加^[10]^。一项实体瘤的荟萃分析^[11]^显示，与单一纳武利尤单抗组相比，纳武利尤单抗联合伊匹木单抗组任何级别和3级以上irAEs发生率更高，联合治疗对比单一治疗3级以上肝毒性发生率（10.1% *vs* 1.3%）及胃肠道毒性发生率（9.9% *vs* 1.2%）明显升高。KEYNOTE-598研究结果^[12]^显示，帕博丽珠单抗联合伊匹木单抗对比单一帕博丽珠单抗在NSCLC一线治疗中未提高疗效，但毒性明显增加。两组3级以上irAEs发生率分别为20.2%和7.8%，联合治疗组3级以上肺炎发生率（5.7% *vs* 2.5%）和结肠炎发生率（5.0% *vs* 1.1%）更高。CheckMate-227研究结果^[13]^显示，纳武利尤单抗联合低剂量伊匹木单抗对比化疗在NSCLC一线治疗中获益，且双免疫治疗组TRAEs发生率更低。双免疫组3级以上TRAEs发生率为33%，而化疗组3级以上TRAEs发生率为36%，双免疫治疗组较化疗组骨髓抑制、恶心呕吐等发生率更低，未出现不可预期的严重毒性反应。

### 1.2 PD-L1抑制剂联合CTLA-4抑制剂

一项晚期一线治疗NSCLC的研究^[14]^显示，度伐利尤单抗组、度伐利尤单抗联合Tremelimumab组和化疗组的3级以上TRAEs发生率分别为14.9%、22.9%和33.8%。单免疫组及双免疫组较化疗组骨髓抑制的发生率明显降低，度伐利尤单抗联合Tremelimumab组腹泻的发生率（2.4%）较单免疫组（0.5%）及化疗组（0.6%）明显升高。

综上，PD-1/PD-L1抑制剂联合CTLA-4抑制剂治疗较单一免疫治疗TRAEs发生率升高但低于化疗，且明显降低了骨髓抑制及恶心呕吐的发生率，这种去化疗的治疗模式可以提高患者生活质量、增加患者治疗依从性，在未来肺癌一线治疗中大有前景。但双免疫治疗中严重的结肠炎、肝脏毒性、肺炎发生率较高，治疗过程中应密切监测。低剂量、加大间隔给药可能在维持抗肿瘤活性的同时降低不良反应的发生率和严重程度^[15]^，未来需要进一步探索双免疫治疗的最佳给药剂量、给药频率，以最大限度地提高疗效并降低毒性。

## 2 PD-1/PD-L1抑制剂联合化疗

化疗药物可直接或间接刺激免疫应答、增加肿瘤免疫原性，进而增加免疫治疗疗效。目前，PD-1/PD-L1抑制剂联合化疗已在多项临床试验中显示出良好的缓解率和生存获益，对于NSCLC，PD-1/PD-L1抑制剂联合化疗是晚期一线更有效的选择。

### 2.1 PD-1抑制剂联合化疗

一线治疗非鳞NSCLC的Keynote189研究^[16]^显示，帕博丽珠单抗联合化疗对比单独化疗3级以上所有归因的不良反应发生率为71.9%和66.8%，两组发生率较高的3级以上不良反应主要为贫血（18.3% *vs* 15.8%）和中性粒细胞减少（16.0% *vs* 12.4%）。联合治疗组3级以上irAEs发生率为10.9%，发生率较高的为肺炎（3.0%）、皮肤毒性（2.2%）、结肠炎（1.5%）和肾炎（1.5%）。

### 2.2 PD-L1抑制剂联合化疗

一线治疗NSCLC的IMpower130研究结果显示，阿替利珠单抗联合化疗组对比单独化疗组，3级以上TRAEs的发生率分别为74.9%和60.7%，主要为中性粒细胞减少（32.0% *vs* 28.0%）和贫血（29.0% *vs* 20.0%），联合治疗组中irAEs发生率为45.0%，最常见的是皮疹（24.0%）和甲状腺功能减退症（15.0%）^[17]^。

### 2.3 双免疫治疗联合化疗

一项纳武利尤单抗与低剂量伊匹木单抗联合化疗（2个周期）一线治疗转移性NSCLC患者的研究^[18]^显示，双免疫联合化疗组与化疗组3级以上TRAEs发生率分别为47%和38%，其中最常见的是中性粒细胞减少（7% *vs* 9%）、贫血（6% *vs* 14%）和腹泻（4% *vs* 1%）。短周期的化疗会增加一些不良反应，但总的来说与既往双免疫治疗研究的毒性发生率非常相似^[13]^。

综上，PD-1/PD-L1抑制剂联合化疗较单一化疗的严重TRAEs发生率未显著增加，主要TRAEs仍为骨髓抑制，在治疗过程中需要密切监测患者血象。双免疫治疗基础上增加短周期的化疗未显著增加TRAEs，并可能有助于患者实现早期疾病控制，未来关于ICIs与化疗的最佳联合周期及剂量值得进一步探索，希望能找到更加高效低毒的联合方案。

## 3 PD-1/PD-L1抑制剂联合抗VEGFR单克隆抗体

抗VEGFR单克隆抗体可通过抑制血管内皮生长因子诱导肿瘤血管的正常化，重塑肿瘤微环境来减少免疫抑制途径^[19]^。临床前研究^[20]^表明，ICIs联合抗VEGFR单克隆抗体治疗可促进抗原提呈细胞的成熟和毒性T淋巴细胞的活化和浸润，减少肿瘤组织中髓样来源的抑制细胞和调节性T细胞的浸润，可协同促进抗肿瘤作用。目前，PD-1/PD-L1抑制剂联合抗VEGFR单克隆抗体治疗已在多个临床试验中看到临床获益。

### 3.1 PD-L1抑制剂联合抗VEGFR单克隆抗体

PD-L1抑制剂联合抗VEGFR单克隆抗体在晚期一线肝细胞癌的治疗获得了良好的效果。有研究^[21]^显示，阿替利珠单抗联合贝伐珠单抗组对比单一阿替利珠单抗组3级以上TRAEs发生率分别为20%和5%，联合治疗组最常见的3级以上TRAEs为高血压（5%）和蛋白尿（3%）。在肺癌治疗中，阿替利珠单抗+贝伐珠单抗+卡铂+紫杉醇四药联合也取得良好的效果，并已成为美国国家综合癌症网络（National Comprehensive Cancer Network, NCCN）指南中非鳞NSCLC的一线治疗推荐。有研究^[22]^表明，阿替利珠单抗+贝伐珠单抗+卡铂+紫杉醇治疗组和贝伐珠单抗+卡铂+紫杉醇治疗组3级以上TRAEs发生率分别为59.5%和50.8%，四药联合整体毒性发生率在预期范围内。

### 3.2 PD-1抑制剂联合抗VEGFR单克隆抗体

目前在肺癌治疗中，PD-1抑制剂联合抗VEGFR单克隆抗体的Ⅲ期以上研究结果较少，一项帕博利珠单抗与抗VEGFR单克隆抗体雷莫芦单抗联合治疗的研究^[23]^显示，在未经治疗的晚期NSCLC患者中，3级以上TRAEs发生率为42.3%，其中最常见的是高血压（15.4%），有2例患者发生了急性心肌梗塞（7.7%）。

综上，PD-1/PD-L1抑制剂联合抗VEGFR单克隆抗体总体安全可行，3级以上TRAEs主要是高血压，联合治疗未出现新的及更严重的TRAEs。治疗过程中应重视对血压的监测和控制，对于出血风险及心血管疾病的发生也应提高警惕。

## 4 PD-1/PD-L1抑制剂联合小分子TKIs

### 4.1 PD-1/PD-L1抑制剂联合多靶点小分子TKIs

纳武利尤单抗联合瑞戈非尼治疗经标准治疗失败的后线复治患者的REGONIVO研究^[24]^显示，瑞戈非尼可以改变VEGFR状态、改变巨噬细胞状态，改变调节性T细胞状态，使免疫治疗疗效提高，且没有严重免疫特异性不良反应的增加。这项研究不仅为末线微卫星稳定型肠癌的治疗带来临床获益，也为联合治疗方案提供了新思路。近几年，PD-1/PD-L1抑制剂与多靶点小分子TKIs的联合治疗在多种实体瘤治疗中都观察到了一定效果且安全性可耐受，如帕博丽珠单抗联合仑伐替尼治疗晚期肝细胞癌^[25]^，帕博丽珠单抗联合阿昔替尼治疗晚期肾细胞癌^[26]^等。目前，在晚期肺癌治疗中，PD-1/PD-L1抑制剂与多靶点小分子TKIs联合治疗的方案也在积极探索中，并已观察到了一定临床获益。

#### 4.1.1 PD-1/PD-L1抑制剂联合仑伐替尼

一项帕博丽珠单抗联合仑伐替尼的研究^[27]^显示，3级以上TRAEs总体发生率为67%，其中发生率较高的为高血压（20%）、疲劳（12%）、腹泻（9%）；3级以上irAEs发生率为10%，主要为肾上腺功能减退（1.5%）和结肠炎（1.5%）。

#### 4.1.2 PD-1/PD-L1抑制剂联合安罗替尼

一项信迪利单抗联合安罗替尼作为晚期NSCLC一线治疗的Ⅰ期研究^[28]^显示，3级以上TRAEs发生率为54.5%，除有2例患者发生高血压（9.1%）外，大多数3级以上TRAEs仅发生一次。常见的irAEs是甲状腺功能减退（50.0%）和肺炎（13.6%）。

#### 4.1.3 PD-1/PD-L1抑制剂联合阿帕替尼

一项卡瑞丽珠单抗联合阿帕替尼治疗既往接受过化疗的晚期非鳞NSCLC患者的Ⅱ期研究^[29]^显示，3级以上TRAEs发生率为69.5%，其中最常见的是高血压（18.1%）、手足皮肤反应（13.3%）、γ-谷氨酰转移酶增高（9.5%）；3级以上irAEs发生率为17.1%，其中以免疫相关性肺炎最常见（1.9%）。

综上，PD-1/PD-L1抑制剂与多种多靶点小分子TKIs的联合治疗都显示出了一定临床获益且安全性可耐受，发生的3级以上TRAEs以高血压为主，暂未发现严重免疫特异性不良反应的增加。但目前PD-1/PD-L1抑制剂与多靶点TKIs的联合治疗研究多以Ⅰ期-Ⅱ期为主，未来还需要Ⅲ期临床研究结果对疗效及安全性进一步验证。目前，在治疗过程中需要密切监测各种不良反应，尤其加强对高血压的监测和控制。

### 4.2 PD-1/PD-L1抑制剂联合表皮生长因子受体（epidermal growth factor receptor, EGFR）-TKIs

EGFR-TKIs不仅可以直接抑制肿瘤细胞的生存能力，而且还可以通过下调PD-L1间接增强抗肿瘤免疫力^[30]^。一项评估NSCLC患者一线接受阿替利珠单抗联合厄洛替尼治疗的Ⅰ期研究^[31]^显示，联合治疗未能使患者获益且TRAEs增加，患者3级以上TRAEs发生率为39%，主要为丙氨酸转氨酶升高（7%）、皮疹（7%）和发热（7%）。一项在肺癌治疗中应用度伐利尤单抗联合奥希替尼的Ib期临床试验^[32]^中，由于间质性肺疾病的高发生率而终止了度伐利尤单抗联合奥希替尼治疗。目前，PD-1/PD-L1抑制剂联合EGFR-TKIs未获得明显的临床获益，而联合治疗的间质性肺炎和肝功能异常发生率较高，联合治疗的可行性还需要进一步探索。

## 5 ICIs联合放疗

局部放疗可激活免疫系统，诱导免疫细胞对放疗区域以外的肿瘤细胞进行攻击，还可以促进肿瘤细胞释放肿瘤特异性抗原，提高免疫系统的杀伤能力^[33]^。目前，ICIs联合放疗已经在多项临床试验中显著提升了患者治疗的有效率，提示放疗和ICIs治疗的协同作用。

### 5.1 ICIs联合胸部放疗

一项对于接受帕博丽珠单抗治疗的NSCLC患者研究的二次分析结果^[34]^显示，接受胸部放疗的患者的肺毒性较未接受放疗的发生率高（63% *vs* 40%）。PACIFIC研究^[35]^中，度伐利尤单抗联合放疗组和单独放疗组3级以上任何原因不良事件发生率分别为30.5%和26.1%，其中最常见的为肺炎（3.4%和2.6%）。另有一项回顾性研究^[36]^显示，肺部立体定向放疗（stereotactic body radiation therapy, SBRT）同步ICIs组与单纯SBRT组相比，发生3级以上肺炎的风险更高（10.7% *vs* 0%），双免疫治疗联合SBRT发生任何级别肺炎的风险明显增加（62.5% *vs* 29.2%）。关于免疫治疗与放疗同步还是序贯的问题，有研究^[37]^显示，阿替利珠单抗与放疗同步治疗组对比序贯治疗组的不良反应发生率并没有增加。

### 5.2 ICIs联合颅脑放疗

有研究^[39]^显示，黑色素瘤患者全脑放疗联合ICIs治疗会增加放射性坏死的风险，也有研究^[38]^显示联合治疗未增加脑坏死的风险。一项对非鳞NSCLC患者的回顾性分析^[40]^显示，接受免疫治疗联合颅脑照射的脑转移患者与总体患者相比，总体TRAEs发生率相似（35% *vs* 33%），未观察到脑水肿和放射性脑坏死。

综上，ICIs联合放疗是安全可行的，但对于放射部位的损伤需密切监测，尤其在联合胸部放疗时，需严密监测放射性肺炎的发生。同时，治疗过程中需严格控制“剂量-体积”，以降低放射性坏死的发生风险^[38]^。

## 6 小结和展望

目前临床研究数据显示，PD-1/PD-L1联合治疗整体安全可行。双免疫治疗中严重的结肠炎、肝脏毒性、肺炎发生率较高，PD-1/PD-L1抑制剂联合化疗的主要TRAEs为骨髓抑制，PD-1/PD-L1抑制剂联合抗VEGFR单克隆抗体与PD-1/PD-L1抑制剂联合多靶点TKIs治疗的3级以上TRAEs主要为高血压，PD-1/PD-L1抑制剂联合EGFR-TKIs间质性肺炎和肝功能异常的发生率明显增加，ICIs联合胸部放疗可能会增加放射性肺炎的发生率。在临床实践中，应根据联合治疗不同的毒性谱提前评估相关毒性并制定相应管理策略，对于发生率高的严重毒性重点监测，以便早期发现并及时治疗。

随着免疫治疗的发展，联合用药的方式越来越多样化，由于不同药物可能通过多种机制相互作用，使得联合治疗具有很大的复杂性。ICIs联合治疗如何更合理、有效低毒地进行联合还需要进一步探索。未来，需要更加深入地了解ICIs联合治疗的具体作用机制，针对不同患者个体化设计最佳联合方案、联合方式、联合用药剂量和用药顺序，在减少不良反应的同时实现免疫治疗抗肿瘤效应的最大化，让肿瘤ICIs联合治疗走向精准之路。

## References

[1] Maio M, Grob J, Aamdal S (2015). Five-year survival rates for treatment-naive patients with advanced melanoma who received ipilimumab plus dacarbazine in a phase Ⅲ trial. J Clin Oncol.

[2] Horn L, Spigel D, Vokes E (2017). Nivolumab versus docetaxel in previously treated patients with advanced non-small-cell lung cancer: two-year outcomes from two randomized, open-label, phase Ⅲ trials (CheckMate 017 and CheckMate 057). J Clin Oncol.

[3] Horn L, Mansfield A, Szczęsna A (2018). First-line atezolizumab plus chemotherapy in extensive-stage small-cell lung cancer. N Engl J Med.

[4] Bellmunt J, De Wit R, Vaughn D (2017). Pembrolizumab as second-line therapy for advanced urothelial carcinoma. N Engl J Med.

[5] Qin SK, Guo J, Li J (2019). Management of immune checkpoint inhibitor-related toxicity of the Chinese Society of Clinical Oncology (CSCO).

[6] Almutairi A, Mcbride A, Slack M (2020). Potential immune-related adverse events associated with monotherapy and combination therapy of ipilimumab, nivolumab, and pembrolizumab for advanced melanoma: a systematic review and meta-analysis. Front Oncol.

[7] Pillai R, Behera M, Owoniakoko T (2018). Comparison of the toxicity profile of PD-1 versus PD-L1 inhibitors in non-small cell lung cancer: A systematic analysis of the literature. Cancer.

[8] Chang C, Park H, Malone D (2020). Immune checkpoint inhibitors and immune-related adverse events in patients with advanced melanoma: a systematic review and network meta-analysis. JAMA Netw Open.

[9] Curran M, Montalvo W, Yagita H (2010). PD-1 and CTLA-4 combination blockade expands infiltrating T cells and reduces regulatory T and myeloid cells within B16 melanoma tumors. Proc Natl Acad Sci U S A.

[10] Larkin J, Chiarion-Sileni V, Gonzalez R (2015). Combined nivolumab and ipilimumab or monotherapy in untreated melanoma. N Engl J Med.

[11] Xing P, Zhang F, Wang G (2019). Incidence rates of immune-related adverse events and their correlation with response in advanced solid tumours treated with NIVO or NIVO+IPI: a systematic review and meta-analysis. J Immunother Cancer.

[12] Boyer M, Şendur M, Rodríguez-Abreu D (2021). Pembrolizumab plus ipilimumab or placebo for metastatic non-small-cell lung cancer with PD-L1 tumor proportion score ≥ 50%: randomized, double-blind phase Ⅲ KEYNOTE-598 study. J Clin Oncol.

[13] Ramalingam SS, Ciuleanu TE, Pluzanski A (2020). Nivolumab + ipilimumab versus platinum-doublet chemotherapy as first-line treatment for advanced non-small cell lung cancer: Three-year update from CheckMate 227 Part 1. J Clin Oncol.

[14] Rizvi N, Cho B, Reinmuth N (2020). Durvalumab with or without tremelimumab *vs* standard chemotherapy in first-line treatment of metastatic non-small cell lung cancer: the MYSTIC Phase 3 randomized clinical trial. JAMA Oncol.

[15] Long G, Atkinson V, Cebon J (2017). Standard-dose pembrolizumab in combination with reduced-dose ipilimumab for patients with advanced melanoma (KEYNOTE-029): an open-label, phase 1b trial. Lancet Oncol.

[16] Gadgeel S, Rodriguez-Abreu D, Speranza G (2020). Updated analysis from KEYNOTE-189: pembrolizumab or placebo plus pemetrexed and platinum for previously untreated metastatic nonsquamous non-smallcell lung cancer. J Clin Oncol.

[17] West H, McCleod M, Hussein M (2019). Atezolizumab in combination with carboplatin plus nab-paclitaxel chemotherapy compared with chemotherapy alone as first-line treatment for metastatic non-squamous non-small-cell lung cancer (IMpower130): a multicentre, randomised, open-label, phase 3 trial. Lancet Oncol.

[18] Reck M, Ciuleanu TE, Dols MC (2020). Nivolumab (NIVO) + ipilimumab (IPI) + 2 cycles of platinum-doublet chemotherapy (chemo) *vs* 4 cycles chemo as first-line (1L) treatment (tx) for stage IV/recurrent non-small cell lung cancer (NSCLC): CheckMate 9LA. J Clin Oncol.

[19] Datta M, Coussens L, Nishikawa H (2019). Reprogramming the tumor microenvironment to improve immunotherapy: emerging strategies and combination therapies. Am Soc Clin Oncol Educ Book.

[20] Shigeta K, Datta M, Hato T (2020). Dual programmed death receptor-1 and vascular endothelial growth factor receptor-2 blockade promotes vascular normalization and enhances antitumor immune responses in hepatocellular carcinoma. Hepatology.

[21] Lee M, Ryoo B, Hsu C (2020). Atezolizumab with or without bevacizumab in unresectable hepatocellular carcinoma (GO30140): an open-label, multicentre, phase 1b study. Lancet Oncol.

[22] Socinski MA, Mok TS, Nishio M (2020). Abstract CT216: IMpower150 final analysis: Efficacy of atezolizumab (atezo) + bevacizumab (bev) and chemotherapy in first-line (1L) metastatic nonsquamous (nsq) non-small cell lung cancer (NSCLC) across key subgroups. Cancer Res.

[23] Herbst R, Arkenau H, Bendell J (2021). Phase 1 expansion cohort of ramucirumab plus pembrolizumab in advanced treatment-naive NSCLC. J Thorac Oncol.

[24] Fukuoka S, Hara H, Takahashi N (2020). Regorafenib plus nivolumab in patients with advanced gastric or colorectal cancer: an open-label, dose-escalation, and dose-expansion phase Ib trial (REGONIVO, EPOC1603). J Clin Oncol.

[25] Finn R, Ikeda M, Zhu A (2020). Phase Ib study of lenvatinib plus pembrolizumab in patients with unresectable hepatocellular carcinoma. J Clin Oncol.

[26] Rini B, Plimack E, Stus V (2019). Pembrolizumab plus axitinib versus sunitinib for advanced renal-cell carcinoma. N Engl J Med.

[27] Taylor M, Lee C, Makker V (2020). Phase IB/Ⅱ trial of lenvatinib plus pembrolizumab in patients with advanced renal cell carcinoma, endometrial cancer, and other selected advanced solid tumors. J Clin Oncol.

[28] Chu T, Zhong R, Zhong H (2021). Phase 1b study of sintilimab plus anlotinib as first-line therapy in patients with advanced NSCLC. J Thorac Oncol.

[29] Zhou C, Wang Y, Zhao J (2021). Efficacy and biomarker analysis of camrelizumab in combination with apatinib in patients with advanced nonsquamous NSCLC previously treated with chemotherapy. Clin Cancer Res.

[30] Chen N, Fang W, Zhan J (2015). Upregulation of PD-L1 by EGFR activation mediates the immune escape in *EGFR*-driven NSCLC: implication for optional immune targeted therapy for NSCLC patients with EGFR mutation. J Thorac Oncol.

[31] Ma BBY, Rudin CM, Cervantes A (2016). Preliminary safety and clinical activity of erlotinib plus atezolizumab from a phase Ib study in advanced NSCLC. Ann Oncol.

[32] Oxnard G, Yang J, Yu H (2020). TATTON: a multi-arm, phase Ib trial of osimertinib combined with selumetinib, savolitinib, or durvalumab in *EGFR*-mutant lung cancer. Ann Oncol.

[33] Citrin D (2017). Recent developments in radiotherapy. N Engl J Med.

[34] Shaverdian N, Lisberg A, Bornazyan K (2017). Previous radiotherapy and the clinical activity and toxicity of pembrolizumab in the treatment of non-small-cell lung cancer: a secondary analysis of the KEYNOTE-001 phase 1 trial. Lancet Oncol.

[35] Antonia SJ, Vi l legas A, Daniel D (2018). Overal l survival with durvalumab after chemoradiotherapy in stage Ⅲ NSCLC. N Engl J Med.

[36] Tian S, Switchenko J, Buchwald Z (2020). Lung stereotactic body radiation therapy and concurrent immunotherapy: a multicenter safety and toxicity analysis. Int J Radiat Oncol Biol Phys.

[37] Lin S, Lin Y, Yao L (2020). Phase Ⅱ trial of concurrent atezolizumab with chemoradiation for unresectable NSCLC. J Thorac Oncol.

[38] Fried DV, Person O, Mavroidis P (2017). Dose immunotherapy alter the dose/volume/outcome relationship in the normal brain?. Int I Radiat Oncol Biol Phys.

[39] Fang P, Jiang W, Allen P (2017). Radiation necrosis with stereotactic radiosurgery combined with CTLA-4 blockade and PD-1 inhibition for treatment of intracranial disease in metastatic melanoma. J Neurooncol.

[40] Crino L, Bronte G, Bidoli P (2019). Nivolumab and brain metastases in patients with advanced non-squamous non-small cell lung cancer. Lung Cancer.

